# PRIME-Teen—Treatment Persistence and Outcomes Associated with CGRP Monoclonal Antibodies Compared with Conventional Oral Preventives in Adolescents with High-Burden Migraine: An Exploratory Real-World Analysis from the German Pain e-Registry (GPeR)

**DOI:** 10.3390/jcm15051976

**Published:** 2026-03-04

**Authors:** Michael A. Überall

**Affiliations:** IFNAP—Private Institute of Neurological Sciences, Nordostpark 51, 90411 Nürnberg, Germany; michael.ueberall@ifnap.de; Tel.: +49-911-21773760; Fax: +49-911-21773761

**Keywords:** migraine, pediatric migraine, adolescents, migraine prevention, CGRP monoclonal antibodies, real-world evidence, treatment persistence, effectiveness, German Pain e-Registry

## Abstract

**Background**: Adolescent migraine is highly prevalent and associated with substantial functional and psychosocial burden. Conventional oral preventives are widely used off-label with limited pediatric efficacy and frequent tolerability problems. Real-world data on calcitonin gene-related peptide (CGRP) monoclonal antibodies in adolescents are scarce. **Methods**: We conducted an exploratory, retrospective cohort analysis of depersonalized routine-care data from adolescents with migraine in the German Pain e-Registry. Patients were eligible if they had at least one 6-month episode with high-evidence conventional oral preventives (HECP) and one 6-month episode with a CGRP monoclonal antibody (CGRP-mAb), each with baseline and follow-up documentation, enabling intra-individual descriptive comparisons. The primary endpoint was a pragmatic composite of 6-month treatment persistence and ≥50% reduction in monthly migraine days (MMD). Secondary outcomes included MMD, MMD with acute medication (MMDAM), migraine-related sick-leave days (MMSLD), disability (MIDAS), and patient-reported psychosocial outcomes. **Results**: A total of 422 adolescents contributed 1448 HECP and 422 CGRP-mAb episodes. Premature discontinuation occurred in 68.8% (HECP) and 11.9% (CGRP-mAb) of episodes; corresponding 6-month persistence was 30.6% and 88.2%, respectively. Mean MMD decreased from 11.7 to 9.4 during HECP episodes and from 11.6 to 4.4 during CGRP-mAb episodes. A ≥50% MMD reduction occurred in 25.4% (HECP) and 70.9% (CGRP-mAb) of episodes; the composite endpoint was met in 23.7% and 69.9%, respectively. CGRP-mAb episodes were associated with numerically larger improvements across secondary outcomes. **Conclusions**: In this high-burden adolescent cohort, CGRP-mAb treatment episodes were associated with higher persistence and broader improvements than prior conventional preventive episodes. Given the retrospective, non-randomized, sequential design, these findings are hypothesis-generating and do not constitute evidence of comparative effectiveness. Controlled pediatric trials and long-term safety studies are warranted.

## 1. Introduction

Migraine is one of the most prevalent neurological disorders in childhood and adolescence and constitutes a major cause of functional impairment and disability during the school-age years [1,2]. Epidemiological studies report lifetime prevalence rates of up to one-third of adolescents, with onset typically occurring prior to adulthood and with notable acceleration during early adolescence, particularly among females following pubertal transition [3,4]. The burden of pediatric migraine extends beyond episodic pain: recurrent attacks lead to absenteeism, impaired academic performance, concentration difficulties, irritability, sleep disruption, reduced engagement in extracurricular and social activities, emotional dysregulation, and deterioration in physical and psychosocial quality of life [5,6,7,8]. These multidimensional consequences arise during a critical developmental window when adolescents are expected to consolidate cognitive skills, establish autonomy, develop social identity, and acquire foundational emotional competencies. Accordingly, recurrent migraine is not merely a source of discomfort but a condition with the potential to alter developmental trajectories and long-term life chances.

The neurobiological underpinnings of pediatric migraine are multifaceted, encompassing activation of trigeminovascular pathways, neurogenic inflammation, altered central pain modulation, and interactions between genetic vulnerabilities and environmental triggers. However, migraine phenotypes in adolescents often diverge from adult disease, with greater symptom variability, more frequent involvement of non-headache features such as gastrointestinal disturbance, fatigue, dizziness, mood changes, and sleep disruption, and increased susceptibility to stress, sensory overload, and hormonal fluctuations. The broader psychosocial environment—including academic pressure, peer evaluation, and emerging self-concept—can amplify symptoms, perpetuate stress responses, and contribute to a cycle of disability.

Despite the high prevalence and profound burden of pediatric migraine, preventive treatment options remain strikingly limited. Most conventional oral preventive agents used in pediatric practice—including the high-evidence conventional prophylactics (HECP) such as beta-blockers, tricyclic antidepressants, flunarizine, topiramate, serotonin noradrenaline reuptake inhibitors, and valproic acid—are prescribed off-label, with limited pediatric efficacy data and well-documented tolerability concerns. The CHAMP trial, which evaluated amitriptyline and topiramate in children and adolescents, demonstrated no superiority over placebo in reducing headache frequency, yet higher rates of adverse effects and treatment discontinuation [9]. Similar findings have emerged in pediatric studies of other conventional agents, suggesting that pharmacotherapies extrapolated from adult practice may not adequately address pediatric migraine biology or treatment needs [10,11,12,13,14]. Tolerability is a major barrier to effective preventive therapy in adolescents. Many conventional agents exert widespread cognitive, behavioral, and metabolic effects, including sedation, cognitive slowing, weight gain, mood instability, irritability, exercise intolerance, and orthostatic symptoms [10,11,12,13,14]. These adverse effects undermine adherence and compromise academic and social functioning, further exacerbating the developmental impact of migraine. Adolescents may be particularly vulnerable to such effects due to ongoing neurobiological maturation, heightened sensitivity to centrally active pharmacological agents, evolving hormonal processes, and the psychosocial relevance of performance, appearance, and peer acceptance.

Despite the fact the HECP are widely used and guideline-recommended—based on the available evidence—their antimigraine effects were discovered by chance, and their mode of action leading to a decrease in migraine frequency remains poorly understood. This contrasts with the calcitonin gene-related peptide (CGRP) monoclonal antibodies (mAbs), which represent a novel class of migraine preventive therapies with a mechanism-based approach that directly targets migraine-specific pathways. CGRP plays a central role in trigeminovascular activation, vasodilation, peripheral and central sensitization, and neurogenic inflammation [15,16]. Unlike the HECP, CGRP-mABs lack broad central nervous system activity and thus have fewer psychotropic or metabolic side effects. Extensive clinical studies in adults have demonstrated not only a significantly faster onset of action but also significant reductions in migraine frequency, improvements in functional outcomes, and, most importantly, a favorable tolerability relative to traditional therapies, resulting in superior treatment persistence [17,18,19,20,21].

Pediatric evidence for CGRP-mABs is emerging [22]. The SPACE-EM randomized controlled trial—reported so far only in the form of a congress presentation [23]—demonstrated significant reductions in migraine frequency with fremanezumab in children aged six to seventeen, with a tolerability profile comparable to adults, adverse drug reactions comparable to placebo, and—most importantly—no safety signals associated with growth, development, or cardiovascular function. Based on these data, fremanezumab has been approved in the United States for preventive treatment of episodic migraine in children aged ≥6 years with a body weight of ≥45 kg on 5 August 2025 [24]. This approval represents an important regulatory milestone for the US, but neither resolves the broader evidence gap for adolescents with high-frequency episodic or even chronic migraine, multiple prior preventive failures and substantial functional and psychosocial impairment, nor helps those migraine adolescents in the rest of the world, where none of the CGRP-mABs is currently approved for patients younger than 18 years of age, and where the use of these therapies in adolescents or even children remains strictly off-label. At the same time, the HECP recommended and used in pediatric migraine are off-label or supported only by weak pediatric evidence, placing clinicians in the paradoxical situation of having to choose between older yet poorly documented medications with burdensome side effects and newer, mechanism-based therapies with strong adult evidence but limited pediatric data lacking formal approval.

This regulatory context reflects the limited availability of pediatric trial data for many pharmacologicals, not only preventive migraine therapies. Studies across medical specialties indicate that up to 90% of pediatric prescriptions globally are off-label, particularly in neurology, psychiatry, oncology, and critical care, where evidence is scarce and therapeutic urgency is high. Consequently, the absence of regulatory approval for pediatric use does not necessarily reflect the absence of clinical validity but rather of dedicated pediatric trials or, as in the case of CGRP-mABs, delayed activities of regulatory authorities.

Real-world evidence provides essential complementary insights to controlled trials by characterizing treatment persistence, functional outcomes, tolerability, and patient-reported experiences in unselected populations. Given the limitations of HECP and the emerging potential of CGRP-mABs, evaluation of real-world performance in adolescents is timely and necessary.

The study presented here analyzed real-world data from the German Pain e-Registry (GPeR), a large nationwide registry collecting standardized, routine-care data from patients with chronic or difficult-to-treat pain. The objective of the present analysis was to describe and explore associations between treatment persistence, migraine-related outcomes, and preventive treatment type in adolescents with high-burden migraine treated under routine-care conditions. The analysis was designed to enable intra-individual descriptive comparisons between conventional oral preventive treatment episodes and subsequent CGRP monoclonal antibody episodes. Given the retrospective, non-randomized, and sequential nature of the data, the study was not intended to establish comparative effectiveness or causal relationships, but to generate hypotheses and inform the design of future controlled pediatric studies.

## 2. Methods

This study is a retrospective, exploratory analysis of fully depersonalized routine care data from the German Pain e-Registry (GPeR), a web-based, non-interventional registry designed to harmonize and aggregate information on real-world pain care in outpatient practices across Germany [25]. Within GPeR, physicians enter structured clinical information on diagnosis, treatment, and courses, while patients complete individualized electronic questionnaires of scientifically validated and expert-recommended instruments on symptoms, disability, and health-related quality of life. All data are collected as part of daily routine care and tailored to the specific needs of individual treatment cases to fulfill both national data-protection and social-law regulations. Participation in and reporting to the GPeR neither influences diagnostic nor therapeutic decisions, nor does it force physicians to use specific instruments. Participation is voluntary and free of charge for patients, independent of their health insurance status.

For the present analysis, data of adolescents aged 12–17 years with a physician diagnosis of migraine were identified at the cut-off date of 30 June 2024.

Datasets of patients were eligible if they had at least one documented 6-month HECP treatment episode and at least one documented 6-month treatment episode with a CGRP-mAB. For each qualifying episode, baseline documentation at or immediately before treatment initiation and at least one follow-up documentation (latest six months thereafter) was required. Each adolescent contributed one or more treatment episodes with HECP and one with CGRP-mABs, and comparisons between treatment types were based on these episodes.

HECP episodes comprised treatment with beta-blockers (BBL), amitriptyline (TCA), flunarizine (FLU), topiramate (TPM), serotonin–noradrenaline reuptake inhibitors (SNRI), or valproate (VPA). The choice of preventive agent, dosing, treatment duration, and switching strategy was entirely at the discretion of a shared decision process between patients and their physicians and followed clinical needs and national standards of care. The GPeR does not constrain indications or prescribe treatment sequences, and both HECP- and CGRP-targeted preventive treatments were off-label in the adolescent population.

The primary endpoint was a composite of two clinically central aspects of migraine prevention. First, the treatment episode had to be continued over the entire 6-month observation period without discontinuation due to adverse drug reactions (ADRs) or insufficient efficacy. Second, the patient had to achieve a reduction in monthly migraine days (MMD) of at least 50% from baseline to the end of month six. Data on MMDs were derived from patient-reported information on migraine days in the respective observation window. Monthly migraine days with acute medication use (MMDAM) and monthly migraine-related school or work disability days (MMSLD) were assessed in an analogous manner. This composite endpoint was chosen as a pragmatic, clinically motivated exploratory measure and has not been formally validated in pediatric populations. It was not intended as a surrogate for comparative effectiveness.

Secondary endpoints included absolute and relative changes in MMD, MMDAM, and MMSLD from baseline to month six, as well as the distribution of patients across migraine frequency categories [chronic migraine (CM: ≥15 headache days/month with ≥8 migraine days), high-frequency episodic migraine (HFEM: 8–14 MMD), low-frequency episodic migraine (LFEM: 4–7 MMD), and very low-frequency episodic migraine (VLFEM: ≤3 MMD)] at baseline and follow-up. Disability and functional impact were assessed with the migraine disability assessment (MIDAS); migraine-related sleep impairments were evaluated via subitem #6 of the modified pain disability index (mPDI6); health-related quality of life was measured with the physical and mental component scores of the VR-12; analyses of the degree of migraine-related depression, anxiety and stress were performed based on data gathered with the depression, anxiety and stress scale (DASS-21), and the Marburg questionnaire on habitual health findings (MQHHF) has been used for the evaluation of the general well-being of patients.

A “6-month treatment episode” was defined as a documented treatment period with a specific preventive agent that spanned up to six consecutive months and for which baseline documentation at or immediately prior to treatment initiation and at least one follow-up assessment within the subsequent six months were available. Episodes did not necessarily imply continuous exposure at a stable target dose throughout the entire period.

For the primary endpoint, the proportion of patients who continued their preventive treatment over the 6-month evaluation period was assessed (component #1 of the PE), and for the MMD (PE component #2), absolute and relative changes were calculated, and the proportions of patients with a clinically meaningful improvement vs. baseline (≥50%) were determined. For all other parameters, absolute and relative changes were calculated and the proportions of patients with improvement vs. baseline were calculated (Annotation: In line with the relative improvements rates of other parameters, a reduction of ≥50% was also considered for the MIDAS as a clinically meaningful improvement, consistent with thresholds commonly used in adult migraine research, while acknowledging that pediatric-specific minimal clinically important differences have not yet been formally established.). Demographic and clinical baseline as well as prior preventive treatment data were summarized using appropriate descriptive statistics.

Continuous variables were characterized by mean, standard deviation (SD), median, and range. Categorical variables were summarized as absolute and relative (if necessary adjusted) frequencies. Differences in continuous changes between CGRP-mABs episodes and conventional preventive episodes were evaluated using student’s *t*-test. For dichotomous endpoints, such as achievement of the primary composite endpoint or a distinct improvement in a given measure, chi-square analyses (with Edward’s correction), odds ratios (ORs) and relative risks (RR)—both with 95% confidence intervals (CI)—were calculated and reported for descriptive purposes only and should not be interpreted as estimates of comparative treatment effect. From responder proportions, number-needed-to-treat (NNT) or number-needed-to-harm (NNH) values were derived to quantify the clinical benefit/risks of CGRP-mABs compared with HECP. Finally, effect size (ES) measures (e.g., Cohen’s d and phi-coefficient) were used to gain insight into the clinical relevance of biometrical differences found.

Missing data in follow-up variables were handled using last observation carried forward (LOCF) or baseline observation carried forward (BOCF) imputation, consistent with established GPeR methodology and under assumptions of missing at random (MAR) and missing not at random, respectively (MNAR). For the primary composite endpoint, which addressed the primary exploratory objective of this analysis, the family-wise type I error rate was controlled using a Bonferroni adjustment to account for multiple testing within the primary endpoint family. In contrast, secondary and additional outcomes were analyzed in an exploratory manner without formal adjustment for multiplicity, and corresponding *p*-values are therefore interpreted descriptively. All statistical tests were two-sided. In this non-interventional setting, *p*-values are presented as measures of the strength of evidence rather than as strict decision thresholds.

All analyses were conducted retrospectively using fully depersonalized registry data. GPeR operates in accordance with the principles of the Declaration of Helsinki and European data protection legislation. Participation in the registry and the scientific use of depersonalized routine care data are covered by the patients’ and, for minors, their legal guardians’ informed consent obtained prior to GPeR participation in pain centers and again at first use of the electronic platform.

The study concept and the evaluation of anonymized data of the GPeR were reviewed and approved by the steering committees of the German Pain Association and the German Pain League, with the latter paying particular attention to ensuring that patient rights are upheld within the framework of this study. After reviewing the study protocol, the Ethics Board of the German Pain League confirmed that neither a formal ethics approval was required for the retrospective, non-interventional evaluation of fully anonymized routine care data nor any additional individual consent.

Prior to data extraction and analysis, this study was entered in the online register of the European Medicines Agency (EMA) for non-interventional studies (ENCEPP; EU PAS Identifier: EUPAS1000000879) and thus made public.

## 3. Results

A total of 422 adolescents contributed information for this analysis. Demographic and baseline migraine characteristics are shown in Table 1. Average age at treatment initiation with HECP/CGRP was 13.0/15.5 years (*p* < 0.001), and 65.6% were female. Due to the sequential course of the preventive treatment strategies, migraine duration was significantly longer for CGRP vs. HECP episodes (5.0 vs. 2.3 years; *p* < 0.001). The proportion of patients who reported suffering from migraine with aura was numerically higher for CGRP vs. HECP episodes (17.8 vs. 15.2%; *p* = 0.307). Use of acute migraine medication, as well as experience with non-pharmacological preventive measures, correlated with the longer migraine duration.

Baseline migraine-related burden was substantial, but comparable for both treatment cohorts. Mean (SD) MMDs were 11.7 (5.5) vs. 11.6 (5.8) for HECP vs. CGRP treatment episodes (*p* = 0.673), as well as MMDAM [11.4 (5.6) vs. 11.0 (5.5); *p* = 0.273] and MMSLD [9.6 (5.0) vs. 9.3 (5.0); *p* = 0.322]. Average migraine pain intensity was 78.7 vs. 79.2 mm VAS, and MIDAS scores were with mean (SD) values of 47.2 (25.1) vs. 48.4 (24.1; *p* = 0.295) in the severe range. Sleep disturbances (38.2 vs. 39.4 mm VAS; *p* = 0.619), as well as migraine-related interference with physical (41.0 vs. 40.8, *p* = 0.765) and mental QoL (46.0 vs. 45.3; *p* = 0.358) were moderate. In contrast, psychological burden was substantial, with similar proportions of patients showing strong-to-severe levels of depression (23.7 vs. 26.1%; *p* = 0.535), anxiety (72.7 vs. 73.5%; *p* = 0.936), and stress (20.4 vs. 20.6%; *p* = 1.000). Consequently, 50.2 vs. 53.1% of patients treated with HECP vs. CGRP-mABs reported severe impairments of their general well-being (*p* = 0.595).

Overall, 1448 treatment episodes with HECP were identified and selected for this analysis. Sequential switching was common, and exposure to HECP was extensive, reflecting recurrent treatment failure. Adolescents documented an average (SD) experience with 3.6 (1.1) different agents, and more than half of them (54.0%) reported on four or even more different approaches. Among HECP, TCA was the most prevalently documented treatment approach (used by 78.4%), followed by BBL (77.7%), and FLU (71.8%). Not surprisingly, VPA was the least frequently used HECP (due to its complex ADR profile, especially for younger females), but nevertheless reported to be used by 32.2%.

For the comparative cohort, information on 422 treatment episodes with CGRP-mABs was aggregated. Most prevalently used CGRP-mAB was erenumab (42.9%), followed by fremanezumab (36.7%), galcanezumab (14.0%), and eptinezumab (6.4%). With 420 patients, 99.5% of adolescents who reported the use of CGRP-mABs in this analysis fulfilled the expanded US/EU criteria that recommend the use of this novel treatment approach in patients ≤ 18 years of age, if they suffer from ≥8 MMD, report ≥ 10 MMDAM, failed to respond to ≥2 trials with HECPs, and present with a MIDAS score of ≥30 [26,27].

Premature discontinuations were markedly higher under HECP than CGRP-mABs (see Table 2). Overall, 68.8% of episodes were discontinued prematurely with HECP, compared with only 11.9% of CGRP-mAB episodes (OR: 0.06, RR: 0.17; *p* < 0.001; NNH: 2). ADR-related discontinuation occurred in 46.5% of episodes reported with HECP and 7.6% of those with CGRP-mABs (OR: 0.09, RR: 0.16; *p* < 0.001; NNH: 3), whereas discontinuation due to inadequate efficacy occurred in 22.2% and 4.3%, respectively (OR: 0.16, RR: 0.19; *p* < 0.001; NNH: 6).

Average (±SD) MMD decreased from 11.7 ± 5.5 to 9.4 ± 6.4 under HECPs (absolute/relative reduction: 2.4 ± 4.4 days/20.8 ± 32.8%) and from 11.6 ± 5.8 to 4.4 ± 4.0 with CGRP-mABs (absolute/relative reduction: 7.2 ± 5.3 days/61.0 ± 28.6%; see Table 3). Any improvement vs. baseline was seen in 31.5% of patients treated with HECP, compared to 89.6% treated with CGRP-mABs in this sequential cohort and observational setting (OR: 18.7, RR: 2.9; *p* < 0.001; ES: 0.588; NNT: 2). An improvement of the migraine frequency type as a consequence of the preventive treatments with HECP/CGRP resulted in 28.9/81.5% (OR 10.8, RR: 2.8; *p* < 0.001; ES: 0.515; NNT: 2).

MMSLDs improved with HECP/CGRP from 9.6/9.3 at baseline (*p* = 0.322) to 7.6/3.0 at end of month 6 (*p* < 0.001), corresponding to an absolute improvement of 2.0/6.3 days (*p* < 0.001; ES: 0.907) and a relative improvement of 20.5/67.3% (*p* < 0.001; ES: 1.408; Table 4). An improvement of ≥50% vs. baseline was reported by 24.2/81.5% with HECP/CGRP-mABs (OR: 13.8, RR: 3.4; *p* < 0.001; ES: 0.574; NNT: 2).

Reductions in acute medication use (MMDAM) were consistent with MMD and MMSLD improvements. HECPs resulted in a reduction of 2.3 ± 4.2 days, with ≥50% reductions in 25.4% of patients, whereas CGRP-mABs reduced MMDAM by 6.8 ± 5.0 days, with ≥50% reductions in 82.5% of patients (OR: 13.8, RR: 3.3; *p* < 0.001; ES: 0.585; NNT: 2 for the HECP vs. CGRP comparison; Table 5).

Functional disability improved under both treatment modalities, although more substantially with CGRP-mABs compared to HECPs. MIDAS scores decreased by 9.2 ± 17.3 points with HECP (*p* < 0.001; ES: 0.256) and 28.3 ± 20.6 points with CGRP-mABs (*p* < 0.001; ES: 0.951; see Table 6). A ≥50% improvement occurred in 20.4% and 71.3% of patients, respectively (OR: 9.7, RR: 3.4; *p* < 0.001; ES: 0.511; NNT: 2).

Comparable to previous findings, psychological and psychosocial outcomes improved substantially with CGRP-mABs. Under HECP, 26.1 to 31.1% of patients demonstrated any improvement with respect to anxiety, depression, and stress, whereas under CGRP-mABs, 75.8 to 86.7% of patients improved on these measures (Table 7). Physical/mental QoL improved with CGRP-mABs in 89.6/89.3% compared to 31.1/28.7% with HECPs (*p* < 0.001 for both), night sleep improved in 88.9 vs. 29.6% (*p* < 0.001), and general well-being in 93.6 vs. 27.3% of patients who received CGRP-mABs vs. HECPs (*p* < 0.001).

The primary endpoint component #1 (6-month treatment persistence) was reached by 30.6 vs. 88.2% of patients who received HECP vs. CGRP-mABs (OR: 16.9, RR: 2.9; *p* < 0.001; ES: 0.586; NNT: 2), and #2 (≥50% MMD improvement vs. baseline) by 25.4 vs. 70.9%, respectively (OR: 7.2, RR: 2.8; *p* < 0.001; ES: 0.455; NNT: 2; see Table 8). This resulted in a primary composite endpoint achievement in 23.7% of patients with HECPs and 69.9% of patients with CGRP-mABs (OR: 7.5, RR: 3.0; *p* < 0.001; ES: 0.463; NNT: 2). Primary endpoint response rates for different HECPs (Table 9) varied from 33.0% with topiramate to 20.5% with amitriptyline (OR: 1.9, RR: 1.6; *p* = 0.002; ES: 0.136; NNT: 8). Further biometrically significant (yet clinically irrelevant) differences were found in favor of topiramate (33.0%) compared to valproic acid (33.0%; *p* = 0.025; ES: 0.123) and to flunarizine (23.8%; *p* = 0.023; ES: 0.102), and in favor of SNRI (28.9%) vs. amitriptyline (20.5%; *p* = 0.038; ES: 0.094), while all other response differences found among HECPs were insignificant. Achievement rates for individual sc CGRP-mABs (see Table 10) ranged from 64.6% with erenumab to 75.5% with fremanezumab (OR: 1.6, RR: 1.2; *p* = 0.032; ES: 0.117; NNT: 9). Other intracohort comparisons of response rates among the CGRP-mABs were insignificant. Irrespective of the nature of the preventive approaches evaluated (HECP/CGRP), response rates were comparable between male (23.5/69.7%) and female patients (23.8/70.0%; *p* = 1.000).

## 4. Discussion

This large, real-world, retrospective, and exploratory analysis describes associations between CGRP monoclonal antibody treatment episodes and higher treatment persistence as well as broader multidimensional improvements compared with earlier conventional oral preventive episodes in adolescents with migraine. The magnitude and internal consistency of improvements observed under CGRP-targeted therapy are notable, particularly considering the extensive prior treatment exposure and persistent high baseline burden. Given the observational and sequential design, these findings should be interpreted as hypothesis-generating rather than as evidence of comparative effectiveness.

Treatment persistence under conventional preventive therapies was extremely poor, with nearly 70% of episodes discontinued prematurely, consistent with pediatric trial data demonstrating high discontinuation rates due to tolerability and inefficacy [9,10,11,12,13,14]. By contrast, fewer than 12% of CGRP-mAB episodes were discontinued in this analysis. Given that treatment persistence may both influence and reflect treatment response, the higher persistence observed during CGRP-mAB episodes may have contributed to the larger improvements seen, while better tolerability may have reduced the likelihood of discontinuation. However, causal relationships cannot be inferred from the present data.

Improvements in migraine frequency under CGRP-mABs exceeded seven days per month on average and were accompanied by reductions in acute medication use, disability, school absenteeism, psychological burden, and quality-of-life impairment. These effects appear larger than those typically reported in pediatric trials of conventional preventives [9,10,11,12,13,14] and warrant confirmation in prospective controlled pediatric studies.

Equally important are the improvements in psychological functioning and well-being. Migraine in adolescence is rarely a discrete somatic condition; rather, it is a biopsychosocial disorder affecting mood regulation, emotional maturation, academic performance, peer relationships, and identity formation. Improvements across anxiety, depression, stress, sleep, and overall well-being under CGRP-mABs suggest that effective migraine prevention may be associated with improvements across psychosocial domains, although the present design does not allow causal attribution.

The observed associations are consistent with the known mechanism-based targeting of migraine-specific pathways by CGRP-mABs, including trigeminovascular activation, neurogenic inflammation, and central sensitization [15,16]. At the same time, CGRP plays physiological roles in vascular regulation, osteogenesis, and immune modulation [28,29,30], and long-term developmental effects remain insufficiently studied, underscoring the need for continued safety surveillance.

In summary, the data presented here generate hypotheses regarding the potential role of CGRP-targeted therapies in adolescents with high-burden migraine and underscore the need for further controlled and long-term investigations. The present analysis should not be used to guide individual treatment decisions or regulatory evaluations, but to generate testable hypotheses for future pediatric trials.

## 5. Strengths and Limitations

This study has multiple strengths. It includes a large adolescent cohort with high disease burden, extensive treatment histories, and broad psychosocial impairment, representing a clinically challenging population frequently excluded from randomized trials. The paired treatment design allows within-individual comparison of treatment trajectories, reducing heterogeneity attributable to static demographic or clinical factors. Data were collected in routine care, capturing real-world patterns of treatment selection, switching, discontinuation, and functional outcomes across multiple domains.

However, several important limitations must be acknowledged. The exploratory, retrospective, non-randomized, and sequential design precludes causal inference, and the observed associations may be influenced by unmeasured confounding, including confounding by indication, temporal effects, regression to the mean, and expectancy effects. Adolescents who initiated CGRP-mABs may have differed in motivation, expectations, or disease trajectory compared with earlier treatment phases.

Treatment exposures were heterogeneous and clinician-directed, and the registry does not systematically capture dosing, titration schedules, adherence, or non-discontinuation adverse effects, limiting interpretation of tolerability and comparative response patterns.

The primary composite endpoint was a pragmatic exploratory measure and has not been formally validated in pediatric populations, which may limit its interpretability for comparative purposes.

The six-month observation window does not capture long-term durability of benefit or late adverse events, including potential developmental effects on growth, bone health, cardiovascular physiology, or immune function.

Finally, although baseline disease burden prior to each episode was highly similar, the sequential nature of the treatment episodes introduces the possibility of temporal confounding related to maturation, hormonal changes, seasonal variation, or cumulative treatment effects.

## 6. Conclusions

In this exploratory real-world analysis of adolescents with high-burden migraine and multiple prior preventive treatment failures, CGRP monoclonal antibody treatment episodes were associated with higher treatment persistence and broader improvements in migraine frequency, disability, psychosocial burden, sleep, and quality of life than earlier conventional oral preventive episodes.

Because of the retrospective, non-randomized, and sequential nature of the data, causal inference is not possible. The present findings should therefore be regarded as hypothesis-generating and as a basis for the design of prospective, controlled pediatric trials and long-term observational safety programs.

## 7. Transparency/Governance

To ensure analytical independence, data extraction and statistical analyses were performed according to a predefined analysis plan registered in the ENCePP database prior to data access. No external party had access to raw data or any influence on the present analyses or manuscript. All analyses were conducted on fully depersonalized datasets, and results are reported in aggregate form only.

## 8. Plain Language Summary

Adolescents with frequent migraines often try several preventive medicines that may not work well or cause side effects. We analyzed routine-care data from a German registry to explore how teenagers did during earlier conventional preventive treatments and later treatment with CGRP antibodies. We found that, in this group with high disease burden, CGRP antibody treatment periods were linked with fewer migraine days, better functioning, and much higher treatment persistence.

Because this was not a randomized trial and treatments occurred one after another, these results cannot prove that one treatment is better than another. They should be seen as early signals that need to be confirmed in controlled pediatric studies and long-term safety programs.

## Figures and Tables

**Table 1:** Demographic, baseline, and treatment characteristics.

	High-Evidence Conventional Preventives (HECP)		Monoclonal CGRP-mABs		Significance (Effect Size)	
Patients [n (%)]		422	(100.0)		na	
Age [years; mean (SD)]	13.0	(1.7)	15.5	(1.3)	*p* < 0.001	(1.067)
[median (range)]	13	(8–16)	15	(12–17)		
Female gender [n (%)]		277	(65.6)		na	
Migraine with aura [n (%)]	64	(15.2)	75	(17.8)	*p* = 0.307	(0.035)
Migraine duration [years; mean (SD)]	2.3	(1.8)	5.0	(1.7)	*p* < 0.001	(1.048)
Acute medication use [n (%)]	422	(100.0)	422	(100.0)		
Acute medication: migraine specific only [n (%)]	74	(17.5)	93	(22.0)	*p* = 0.101	(0.056)
Non-specific only [n (%)]	211	(50.1)	132	(31.3)	*p* < 0.001	(0.190)
Combination [n (%)]	137	(32.4)	197	(46.7)	*p* < 0.001	(0.145)
Experience with non-pharmaceutical treatment with … PMR [n (%)]	236	(55.9)	309	(73.2)	*p* < 0.001	(0.181)
Biofeedback [n (%)]	196	(46.4)	282	(66.8)	*p* < 0.001	(0.206)
CBT [n (%)]	194	(46.0)	281	(66.6)	*p* < 0.001	(0.208)
Acupuncture [n (%)]	156	(37.0)	265	(62.8)	*p* < 0.001	(0.258)
Sport/physical exercise [n (%)]	248	(58.8)	338	(80.1)	*p* < 0.001	(0.231)
Lifestyle habit change [n (%)]	293	(69.4)	342	(81.0)	*p* < 0.001	(0.134)
Monthly migraine days [MMD; mean (SD)]	11.7	(5.5)	11.6	(5.8)	*p* = 0.673	(0.017)
Monthly migraine days with acute medication [MMDAM; mean (SD)]	11.4	(5.6)	11.0	(5.5)	*p* = 0.273	(0.061)
Monthly migraine-related sick-leave days [MMSLD; mean (SD)]	9.6	(5.0)	9.3	(5.0)	*p* = 0.322	(0.051)
Migraine pain intensity [mm VAS; mean (SD)]	78.7	(15.7)	79.2	(15.7)	*p* = 0.414	(0.008)
Migraine-related disability [MIDAS; mean (SD)]	47.2	(25.1)	48.4	(24.1)	*p* = 0.295	(0.049)
Very severe disability [MIDAS ≥ 41; n (%)]	215	(50.9)	216	(51.2)	*p* = 0.922	(0.002)
Migraine-related impairment of night sleep [mPDI#6, mm VAS; mean (SD)]	38.2	(24.0)	39.4	(23.4)	*p* = 0.619	(0.013)
Physical quality of life [VR-12-PCS; mean (SD)]	41.0	(7.9)	40.8	(8.2)	*p* = 0.765	(0.003)
Mental quality of life [VR-12-MCS; mean (SD)]	46.0	(12.7)	45.3	(12.5)	*p* = 0.358	(0.012)
Strong-to-extreme … depression [DASS-21; n (%)]	100	(23.7)	110	(26.1)	*p* = 0.535	(0.027)
…anxiety [DASS-21; n (%)]	307	(72.7)	310	(73.5)	*p* = 0.936	(0.008)
…stress [DASS-21; n (%)]	86	(20.4)	87	(20.6)	*p* = 1.000	(0.003)
General well-being [MQHHF, NRS35; mean (SD)]	11.1	(7.1)	10.9	(7.0)	*p* = 0.656	(0.010)
Severely impaired general well-being [MQHHF ≤ 10; n (%)]	212	(50.2)	224	(53.1)	*p* = 0.595	(0.029)
HECP treatment with … beta-blockers [n (%)]	328	(77.7)	-		na	
Amitriptyline [n (%)]	331	(78.4)	-		na	
Flunarizine [n (%)]	303	(71.8)	-		na	
Topiramate [n (%)]	194	(46.0)	-		na	
SNRI [n (%)]	156	(37.0)	-		na	
Valproic acid [n (%)]	136	(32.2)	-		na	
Number of HECPs used prior to CGRP [mean (SD)]	3.6	(1.1)	-		na	
1 [n (%)]	7	(1.7)	-		na	
2 [n (%)]	53	(12.6)	-		na	
3 [n (%)]	134	(31.8)	-		na	
4 [n (%)]	139	(32.9)	-		na	
5 [n (%)]	73	(17.3)	-		na	
6 [n (%)]	16	(3.8)	-		na	
CGRP treatment with … Erenumab [n (%)]	-		181	(42.9)	na	
Fremanezumab [n (%)]	-		155	(36.7)	na	
Galcanezumab [n (%)]	-		59	(14.0)	na	
Eptinezumab [n (%)]	-		27	(6.4)	na	
Number of CGRPs used [mean (SD)]	-		1.0	(0.0)	na	

Abbreviations: HECP: high-evidence conventional preventives; CGRP: calcitonin gene-related peptide; mABs: monoclonal antibodies; n: number of patients; %: percentage; SD: standard deviation; PMR: progressive muscle relaxation; CBT: cognitive behavioral therapy; MMD: monthly migraine days; MMDAM: monthly migraine days with acute medication; MMSLD: monthly migraine sick-leave days; VAS: visual analog scale; MIDAS: migraine disability assessment scale; mPDI#6: subitem number 6 of the modified pain disability index; VR-12: veterans RAND short-form quality-of-life questionnaire; PCS: physical component score; MCS: mental component score; DASS: depression, anxiety and stress scale; MQHHF: Marburg questionnaire on habitual health findings; SNRI: serotonin–noradrenaline reuptake inhibitors. na: not applicable.

**Table 2:** Premature treatment discontinuations.

	High-Evidence Conventional Preventives (HECP)		Monoclonal CGRP-mABs	
Patients [n (%)]		422	(100.0)	
Number of treatment episodes evaluated (n)	1448		422	
Premature treatment discontinuations (n)	996		50	
[percent (95% CI)]	68.8 (66.5–71.1)		11.9 (9.1–15.3)	
Odds ratio (95% CI)		0.06 (0.04–0.09)		
Relative risk (95% CI)		0.17 (0.13–0.22)		
Between-cohort significance (effect size)	*p* < 0.001 (0.571)			
Number needed to harm (NNH)	2			
Discontinuations due to drug-related adverse events (n)	674		32	
[percent (95% CI)]	46.5 (44.0–49.1)		7.6 (5.4–10.5)	
Odds ratio (95% CI)		0.09 (0.06–0.14)		
Relative risk (95% CI)		0.16 (0.12–0.23)		
Between-cohort significance (effect size)	*p* < 0.001 (0.433)			
Number needed to harm (NNH)	3			
Discontinuation due to inadequate efficacy (n)	322		18	
[percent (95% CI)]	22.2 (20.2–24.5)		4.3 (2.7–6.6)	
Odds ratio (95% CI)		0.16 (0.10–0.25)		
Relative risk (95% CI)		0.19 (0–12–0.31)		
Between-cohort significance (effect size)	*p* < 0.001 (0.273)			
Number needed to harm (NNH)	6			

Abbreviations: HECP: high-evidence conventional preventives; CGRP: calcitonin gene-related peptide; mABs: monoclonal antibodies; n: number of patients; %: percentage; CI: confidence interval; NNH: number needed to harm.

**Table 3:** Treatment effects on monthly migraine days (MMD) and migraine frequency type.

	High-Evidence Conventional Preventives (HECP)		Monoclonal CGRP-mABs		Between-Cohort Significance (Effect Size)	
Patients [n (%)]		422	(100.0)		na	
Monthly migraine days (MMD) at … baseline [mean (SD)]	11.7	(5.5)	11.6	(5.8)	*p* = 0.673	(0.017)
… end of evaluation period [mean (SD)]	9.4	(6.4)	4.4	(4.0)	*p* < 0.001	(0.715)
Absolute improvement vs. baseline [days; mean (SD)]	2.4	(4.4)	7.2	(5.3)	*p* < 0.001	(0.877)
Relative improvement vs. baseline [percent; mean (SD)]	20.8	(32.8)	61.0	(28.6)	*p* < 0.001	(1.194)
Within-cohort significance (effect size)	*p* < 0.001	(0.273)	*p* < 0.001	(1.022)		
Improvement of migraine frequency (n)	133		378			
[percent (95% CI)]	31.5 (27.3–36.1)		89.6 (86.3–92.1)			
Odds ratio (95% CI)		18.7 (12.9–27.1)				
Relative risk (95% CI)		2.9 (2.6–3.1)				
Between-cohort significance (effect size)	*p* < 0.001 (0.588)					
Number needed to treat (NNT)	2					
Improvement of migraine frequency type (n)	122		344			
[percent (95% CI)]	28.9 (24.8–33.4)		81.5 (77.5–84.9)			
Odds ratio (95% CI)		10.8 (7.9–14.9)				
Relative risk (95% CI)		2.8 (2.4–3.3)				
Between-cohort significance (effect size)	*p* < 0.001 (0.515)					
Number needed to treat (NNT)	2					

Abbreviations: HECP: high-evidence conventional preventives; CGRP: calcitonin gene-related peptide; mABs: monoclonal antibodies; n: number of patients; %: percentage; SD: standard deviation; CI: confidence interval; NNT: number needed to treat.

**Table 4:** Treatment effects on monthly migraine sick-leave days (MMSLD).

	High-Evidence Conventional Preventives (HECP)		Monoclonal CGRP-mABs		Between-Cohort Significance (Effect Size)	
Patients (n)		422	(100.0)		na	
Monthly migraine sick-leave days (MMSLD) at … baseline [mean (SD)]	9.6	(5.0)	9.3	(5.0)	*p* = 0.322	(0.051)
… end of evaluation period [mean (SD)]	7.6	(5.3)	3.0	(3.2)	*p* < 0.001	(0.783)
Absolute improvement vs. baseline [days; mean (SD)]	2.0	(3.7)	6.3	(4.4)	*p* < 0.001	(0.907)
Relative improvement vs. baseline [percent; mean (SD)]	20.5	(32.4)	67.3	(27.6)	*p* < 0.001	(1.408)
Within-cohort significance (effect size)	*p* < 0.001	(0.262)	*p* < 0.001	(1.061)		
Improvement ≥ 50% vs. baseline (n)	102		344			
[percent (95% CI)]	24.2 (20.3–28.5)		81.5 (77.5–84.9)			
Odds ratio (95% CI)		13.8 (9.9–19.3)				
Relative risk (95% CI)		3.4 (2.8–4.1)				
Between-cohort significance (effect size)	*p* < 0.001 (0.574)					
Number needed to treat (NNT)	2					

Abbreviations: HECP: high-evidence conventional preventives; CGRP: calcitonin gene-related peptide; mABs: monoclonal antibodies; n: number of patients; %: percentage; SD: standard deviation; CI: confidence interval; NNT: number needed to treat.

**Table 5:** Treatment effects on monthly migraine days with acute medication (MMDAM).

	High-Evidence Conventional Preventives (HECP)		Monoclonal CGRP-mABs		Between-Cohort Significance (Effect Size)	
Patients (n)		422	(100.0)		na	
Monthly migraine days with acute medication (MMDAM) at … baseline [mean (SD)]	11.4	(5.6)	11.0	(5.5)	*p* = 0.273	(0.061)
… end of evaluation period [mean (SD)]	9.1	(6.1)	4.1	(3.8)	*p* < 0.001	(0.969)
Absolute improvement vs. baseline [days; mean (SD)]	2.3	(4.2)	6.8	(5.0)	*p* < 0.001	(0.856)
Relative improvement vs. baseline [percent; mean (SD)]	20.6	(32.6)	60.6	(28.3)	*p* < 0.001	(1.196)
Within-cohort significance (effect size)	*p* < 0.001	(0.292)	*p* < 0.001	(1.099)		
Improvement ≥ 50% vs. baseline (n)	107		348			
[percent (95% CI)]	25.4 (21.4–29.7)		82.5 (78.6–85.8)			
Odds ratio (95% CI)		13.8 (9.9–19.3)				
Relative risk (95% CI)		3.3 (2.8–3.9)				
Between-cohort significance (effect size)	*p* < 0.001 (0.585)					
Number needed to treat (NNT)	2					

Abbreviations: HECP: high-evidence conventional preventives; CGRP: calcitonin gene-related peptide; mABs: monoclonal antibodies; n: number of patients; %: percentage; SD: standard deviation; CI: confidence interval; NNT: number needed to treat.

**Table 6:** Treatment effects on migraine-related disability (MIDAS).

	High-Evidence Conventional Preventives (HECP)		Monoclonal CGRP-mABs		Between-Cohort Significance (Effect Size)	
Patients [n (%)]		422	(100.0)		na	
Migraine-related disability (MIDAS) at … baseline [mean (SD)]	48.4	(24.1)	47.2	(25.1)	*p* = 0.295	(0.048)
… end of evaluation period [mean (SD)]	39.4	(25.6)	18.9	(16.0)	*p* < 0.001	(0.782)
Absolute improvement vs. baseline [days; mean (SD)]	9.2	(17.3)	28.3	(20.6)	*p* < 0.001	(1.035)
Relative improvement vs. baseline [percent; mean (SD)]	18.9	(30.2)	59.1	(26.4)	*p* < 0.001	(1.294)
Within-cohort significance (effect size)	*p* < 0.001	(0.256)	*p* < 0.001	(0.951)		
Improvement ≥ 50% vs. baseline (n)	86		301			
[percent (95% CI)]	20.4 (16.8–24.5)		71.3 (66.9–75.4)			
Odds ratio (95% CI)		9.7 (7.1–13.4)				
Relative risk (95% CI)		3.4 (2.9–4.3)				
Between-cohort significance (effect size)	*p* < 0.001 (0.511)					
Number needed to treat (NNT)	2					

Abbreviations: HECP: high-evidence conventional preventives; CGRP: calcitonin gene-related peptide; mABs: monoclonal antibodies; n: number of patients; %: percentage; MIDAS: migraine disability assessment scale; SD: standard deviation; CI: confidence interval; NNT: number needed to treat.

**Table 7:** Treatment effects on affective symptomatology (depression, anxiety, stress), physical and mental quality of life, sleep, and general well-being.

	High-Evidence Conventional Preventives (HECP)		Monoclonal CGRP-mABs		Odds Ratio (95% CI)	Relative Risk (95% CI)	Between- Cohort Significance (Effect Size)	
Patients [n (%)]		422	(100.0)		na	na	na	
Any improvement in … depression [DASS-21; n (%)]	123	(29.1)	352	(83.4)	12.2 (8.8–17.0)	2.9 (2.5–3.3)	*p* < 0.001	(0.547)
… anxiety [DASS-21; n (%)]	110	(26.1)	320	(75.8)	8.9 (6.5–12.2)	2.9 (2.5–3.5)	*p* < 0.001	(0.497)
… stress [DASS-21; n (%)]	128	(30.3)	366	(86.7)	15.0 (10.6–21.3)	2.9 (2.5–3.3)	*p* < 0.001	(0.573)
… physical quality of life [VR-12-PCS; n (%)]	131	(31.1)	378	(89.6)	19.1 (13.1–27.7)	2.9 (2.5–3.3)	*p* < 0.001	(0.598)
… mental quality of life [VR-12-MCS; n (%)]	121	(28.7)	377	(89.3)	20.8 (14.3–30.3)	3.1 (2.7–3.6)	*p* < 0.001	(0.617)
…night sleep [mPDI#6; n (%)]	125	(29.6)	375	(88.9)	19.0 (13.1–27.4)	3.0 (2.6–3.5)	*p* < 0.001	(0.603)
…general well-being [MQHHF; n (%)]	115	(27.3)	395	(93.6)	39.1 (25.0–60.9)	3.4 (2.9–4.0)	*p* < 0.001	(0.678)

Abbreviations: HECP: high-evidence conventional preventives; CGRP: calcitonin gene-related peptide; mABs: monoclonal antibodies; CI: confidence interval; n: number of patients; %: percentage; DASS: depression, anxiety, and stress scale; VR-12: veterans RAND short-form quality-of-life questionnaire; PCS: physical component score; MCS: mental component score; mPDI#6: subitem number 6 of the modified pain disability index; MQHHF: Marburg questionnaire on habitual health findings.

**Table 8:** Response to the primary endpoint components and the primary composite endpoint.

	High-Evidence Conventional Preventives (HECP)		Monoclonal CGRP-mABs	
Patients (n)		422	(100.0)	
PEC #1: No premature treatment discontinuation (n)	129		372	
[percent (95% CI)]	30.6 (26.4–35.1)		88.2 (84.7–90.9)	
Odds ratio (95% CI)		16.9 (11.8–24.2)		
Relative risk (95% CI)		2.9 (2.5–3.3)		
Between-cohort significance (effect size)	*p* < 0.001 (0.586)			
Number needed to harm (NNT)	2			
PEC #2: Reduction in monthly migraine days ≥ 50% vs. baseline (MMD; n)	107		299	
[percent (95% CI)]	25.4 (21.4–29.7)		70.9 (66.3–75.0)	
Odds ratio (95% CI)		7.2 (5.3–9.7)		
Relative risk (95% CI)		2.8 (2.3–3.3)		
Between-cohort significance (effect size)	*p* < 0.001 (0.455)			
Number needed to treat (NNT)	2			
Primary endpoint (PE): combination of PEC #1 plus PEC #2 (n)	100		295	
[percent (95% CI)]	23.7 (19.9–28.0)		69.9 (65.4–74.1)	
Odds ratio (95% CI)		7.5 (5.5–10.2)		
Relative risk (95% CI)		3.0 (2.5–3.5)		
Between-cohort significance (effect size)	*p* < 0.001 (0.463)			
Number needed to treat (NNT)	2			

Abbreviations: HECP: high-evidence conventional preventives; CGRP: calcitonin gene-related peptide; mABs: monoclonal antibodies; PEC: primary endpoint component; n: number of patients; %: percentage; CI: confidence interval; NNT: number needed to treat.

**Table 9:** Differential primary composite endpoint response dependent on HECP and gender.

	Patients	Primary Endpoint Reached		Significance (Effect Size)	
	n	n	% (95% CI)		
Beta-blocking agents	328	83	25.3 (20.9–303.)	*p* = 0.027	(0.093)
Amitriptyline	331	68	20.5 (16.5–25.2)		
Flunarizine	303	72	23.8 (19.3–28.9)		
Topiramate	194	64	33.0 (26.8–39.9)		
Serotonin–noradrenaline reuptake inhibitors	156	45	28.9 (22.3–36.4)		
Valproic acid	136	29	21.3 (15.3–29.0)		
Gender: male	145	34	23.5 (17.3–31.0)	*p* = 1.000	(0.000)
Female	277	66	23.8 (19.2–29.2)		
All	422	100	23.7 (19.9–28.0)		

Abbreviations: HECP: high-evidence conventional preventive drugs; n: number of patients; %: percentage; CI: confidence interval.

**Table 10:** Differential primary composite endpoint response dependent of CGRP-mAB and gender.

	Patients	Primary Endpoint Reached		Significance (Effect Size)	
	n	n	% (95% CI)		
CGRP-mAB: Erenumab	181	117	64.6 (57.4–71.2)	*p* = 0.067	(0.108)
Fremanezumab	155	117	75.5 (68.2–81.6)		
Galcanezumab	59	41	69.5 (56.9–79.8)		
Eptinezumab	27	20	74.1 (55.3–86.8)		
Gender: male	145	101	69.7 (61.7–76.6)	*p* = 1.000	(0.000)
Female	277	194	70.0 (64.4–75.1)		
All	422	295	69.9 (65.4–74.1)		

Abbreviations: CGRP: calcitonin gene-related peptide; mABs: monoclonal antibodies; n: number of patients; %: percentage; CI: confidence interval.

## Data Availability

The data presented in this study are not publicly available due to data protection regulations. Aggregated and fully anonymized data may be made available upon reasonable request and subject to approval by the data custodians of the German Pain e-Registry.
